# A Comparative Study of Intrathecal Hyperbaric Bupivacaine With Fentanyl Versus Intrathecal Hyperbaric Bupivacaine With Dexmedetomidine Administered Sequentially for Lower Limb Orthopaedic Surgeries: A Prospective Randomised Double-Blind Study

**DOI:** 10.7759/cureus.73672

**Published:** 2024-11-14

**Authors:** Usha Shukla, Rakesh Bahadur Singh, Kriti Mishra, Vikram S Rathore

**Affiliations:** 1 Department of Anaesthesiology and Critical Care, Uttar Pradesh University of Medical Sciences, Etawah, IND; 2 Department of Anaesthesiology, Uttar Pradesh University of Medical Sciences, Etawah, IND

**Keywords:** 0.5% hyperbaric bupivacaine, fentanyl, intrathecal dexmedetomidine, lower limb surgeries, sequential spinal anesthesia

## Abstract

Background: The subarachnoid block is the predominant and relatively safe approach during lower limb orthopaedic operations. When used as an additive to intrathecal local anaesthetic, both fentanyl and dexmedetomidine can extend the duration of sensory and motor blockade and improve postoperative analgesia.

Objectives: The objective of this study is to assess and compare the efficacy of sequential administration of fentanyl and dexmedetomidine alongside 0.5% hyperbaric bupivacaine intrathecally in lower limb orthopaedic surgeries, focussing on block characteristics, postoperative analgesia as measured by visual analogue scale (VAS) scores, haemodynamic changes, and adverse effects.

Materials and methods: Sixty patients were randomised into two groups of 30 each. Group A received 15 mg of 0.5% hyperbaric bupivacaine followed by 25 µg of fentanyl, whereas group B received 15 mg of 0.5% hyperbaric bupivacaine followed by 5 µg of dexmedetomidine, administered sequentially for spinal anaesthesia.

Results: Patients in group B exhibited a markedly prolonged sensory and motor block duration compared to group A. The duration required to achieve maximum sensory blockage (T6) was reduced significantly in group B (2.40 ± 0.50 minutes) compared to group A (5.03 ± 0.81 minutes). The average duration of sensory regression to S1 was 207.33 ± 20.18 minutes in group B and 146.57 ± 19.01 minutes in group A (p < 0.001). Patients in group B exhibited a markedly prolonged sensory and motor block duration compared to group A. The duration to attain total motor blockage (Modified Bromage 3) was markedly reduced in group B (4.23 ± 0.68 minutes) relative to group A (7.03 ± 0.81 minutes). The overall analgesic required over a 24-hour period and the average VAS ratings were both lower in group B compared to group A. Additionally, patient satisfaction was higher in group B (207.33 ± 20.18 minutes) than in group A (146.57 ± 19.01 minutes) (p < 0.001).

Conclusion: The study concludes that the sequential administration of dexmedetomidine as an adjuvant with the local anaesthetic agent during the subarachnoid block enhances the onset of sensory and motor block, prolongs analgesia, diminishes overall analgesic requirements, increases patient satisfaction, and maintains stable haemodynamics compared to fentanyl. Bradycardia is common with dexmedetomidine.

## Introduction

The subarachnoid block is frequently employed and regarded as a safe anaesthetic method for lower limb orthopaedic procedures [1]. It has numerous advantages, including maintaining patient consciousness during surgery, rapid onset of action, a low failure rate, minimal medication consumption, excellent sensory and motor blockade, cost-effectiveness, and postoperative analgesia. Moreover, it preserves the patient's cognitive condition and standard airway reflexes [2]. Neuraxial analgesia utilising solely local anaesthetics frequently yields inadequate pain reduction and is linked to increased side effects [3]. Bupivacaine, a frequently utilised local anaesthetic in spinal anaesthesia, exhibits more potency than lignocaine and possesses an extended duration of action [4]. Nonetheless, it exhibits a delayed beginning of action and offers a restricted motor blockade. Although bupivacaine has a lengthy duration of action, it does not provide extended postoperative analgesia, requiring the use of adjuvants for sustained pain relief [5]. Adjuvants can alleviate these problems and extend the duration of the anaesthetic effect [6]. Frequently utilised adjuvants include fentanyl, dexmedetomidine, clonidine, ketamine, and neostigmine. Neuraxial opioids seek to provide analgesia equivalent to systemic administration while utilising considerably reduced dosages: fentanyl, predominantly a µ-receptor agonist, and lipophilic opioids exhibit a rapid onset of action (within five minutes) and a duration of three to five hours when delivered intrathecally. A key benefit of "selective spinal analgesia" using fentanyl is the lack of sympathetic blocking and postural hypotension, facilitating early ambulation and mitigating risks such as cardiovascular collapse or seizures, which are significant issues associated with spinal anaesthesia [7,8]. Dexmedetomidine, an imidazole subclass member of α2 adrenergic receptor agonists, is the S-enantiomer of medetomidine. It exhibits a high selectivity for adrenoceptors (α2:α1, 1,600:1) compared to clonidine, categorising it as a full agonist. Dexmedetomidine extends the duration of sensory and motor blocks produced by local anaesthetics, irrespective of the administration route [9]. When injected intrathecally, it exerts effects at both spinal and supraspinal levels by stimulating α2 adrenergic receptors in the spinal cord, diminishing nociceptive signal transmission, decreasing substance P release, and enhancing analgesic efficacy while significantly reducing opioid requirements [10]. This study aimed to assess and compare the effectiveness of fentanyl and dexmedetomidine as sequential adjuvants to intrathecal hyperbaric 0.5% bupivacaine in patients undergoing elective lower limb operations [11].

## Materials and methods

This prospective, randomised trial was undertaken with approval from the Institutional Ethics Committee (ethical clearance number: 41/2022-23). It was registered with the Clinical Trials Registry India (CTRI) under registration number CTRI/2024/05/066585 dated 1 May 2024. The research was conducted from 1 June 2024 to 30 September 2024 at the Uttar Pradesh University of Medical Sciences, Etawah. The study comprises 60 American Society of Anaesthesiologists physical status I and II participants, aged 18-65, scheduled for elective lower limb orthopaedic surgeries. It excludes individuals with hepatic or renal impairment, a history of epilepsy, neuropsychiatric disorders, coagulation abnormalities, cardiac block or dysrhythmias, uncontrolled hypertension or diabetes mellitus, allergies to the study medications, or those who decline participation. The patients were randomly assigned to two equal groups, group A and group B, using sealed opaque envelopes to obscure the allocation sequence. An anaesthesiologist, tasked with producing the drug solution according to the randomisation, opened the sealed envelopes but was not physically engaged in research. The anaesthesiologist doing the block technique and monitoring the study outcomes was unaware of the group treatment, as was the anaesthesiologist tasked with collecting data. In group A, patients were administered 15 mg of 0.5% hyperbaric bupivacaine followed by 25 µg of fentanyl, whereas in group B, patients received 15 mg of 0.5% hyperbaric bupivacaine followed by 5 µg of dexmedetomidine. Patients completed a preoperative evaluation and fasted for eight hours before surgery. They received premedication with 150 mg of ranitidine and 0.5 mg of alprazolam. Airway management apparatus and emergency pharmaceuticals were arranged in the operation room. The intravenous line was established and primed with 500 mL of Ringer's lactate solution before spinal anaesthesia. The patient was attached to a non-invasive blood pressure monitor, an oxygen saturation device, and electrocardiogram (ECG) leads. Preoperative basal vital values were taken. A subarachnoid block was done at the L3-L4 or L4-L5 levels using a midline approach while the patient was seated, utilizing a 26G Quincke spinal needle under stringent aseptic conditions. A total of 15 mg (3 mL) of 0.5% hyperbaric bupivacaine was administered with the operative table in a horizontal position, followed by 0.5 mL of the study drug through the same spinal needle in both groups. The patients were promptly positioned supine, and the time of study drug administration was documented (0 minutes). Haemodynamic monitoring was performed at consistent intervals during the procedure utilising a multi-parameter monitor that displayed heart rate, systolic blood pressure, diastolic blood pressure, mean arterial pressure (MAP), ECG, and oxygen saturation. Monitoring intervals were conducted every five minutes for the initial 15 minutes, every 10 minutes for the subsequent 30 minutes, and every 15 minutes until the conclusion of the surgery. Postoperatively, monitoring occurred at 30, 60, 120, 240, 360, 720, and 1,440 minutes. The complete length of the sensory suppression was documented.

The sensory blockade was assessed using the pinprick method using a blunt-tipped 27G needle, initially at one-minute intervals for five minutes, followed by five-minute intervals for the next 15 minutes. Several characteristics of the blocks were observed. The sensory block was evaluated using a three-point scale: normal sensation (0), loss of pinprick sensation (analgesia) (1), and loss of tactile sensation (anaesthesia) (2). The duration required to achieve maximum sensory blockage (T6) refers to the interval from the administration of the trial drugs to the attainment of maximal sensory blockade (T6). The duration of two-segment sensory regression is the interval from the administration of the study drug until the sensory perception has diminished by two segments. The duration of the sensory blockade is the interval from the administration of the study drug until the patient perceives sensation at the S1 dermatome. The period after receiving the study medication injection until the patient can only move their knee and ankle but not their hip is known as the onset of motor blockage. The Modified Bromage Scale was used to assess motor blockage every minute after the medication administration, where a score of 0 indicates no power impairment and the ability to elevate the leg, 1 indicates unable to move the hip and able to move the knee and ankle, 2 indicates unable to move the hip and knee, and able to move the ankle, and 3 indicates unable to move the hip, knee, and ankle. The time taken to achieve maximal motor blockade is defined as the time between the study drug injection and the maximum motor blockade reached (Modified Bromage 3) [12].

The time period of analgesia is defined as the interval between the introduction of spinal medication and the initial supplementing with rescue analgesia. The pain was assessed using the visual analogue scale (VAS), which ranges from 0 to 10, with 0 indicating no discomfort and 10 representing extreme pain. In both groups, the time following the administration of the block was designated as time zero. The VAS at time zero was the baseline score following the block and was documented for all patients. In the postoperative period, up to 24 hours, if VAS >4, an injection of diclofenac (nonsteroidal anti-inflammatory drugs) in a dose of 1.5 mg/kg was given intramuscularly every eight hours as rescue analgesia [13].

The patient satisfaction scores were evaluated immediately after surgery and 12 hours thereafter based on a scale of 1-7: extremely dissatisfied (1), dissatisfied (2), somewhat dissatisfied (3), undecided (4), somewhat satisfied (5), satisfied (6), and extremely satisfied (7).

Adverse events, including bradycardia (heart rate <50 bpm or a 20% reduction from baseline), hypotension (a 20% decrease in blood pressure from baseline), or an absolute MAP or desaturation event (a decline in mean oxygen saturation of ≥3% for over 10 seconds) occurring during or immediately after the procedure were recorded. Bradycardia was managed with incremental doses of intravenous atropine (0.6 mg). Hypotension was addressed by a bolus of intravenous crystalloids or with incremental intravenous injections of 6 mg mephentermine.

 Statistical analysis

The study's sample size is computed, assuming a 25% difference in haemodynamic stability across the groups. We considered 80% power and calculated the sample size with 95% confidence, resulting in a sample of 26 patients per group. We chose 30 members per group. The sample size calculation is as follows: n = (Zα/β + Ζβ) ² × 2σ² / (µ1 - µ2)². Categorical variables were expressed as counts and percentages (%), whereas continuous variables were reported as means and standard deviations. Quantitative factors were compared between the two groups using the Mann-Whitney U test or unpaired t-test, as applicable. Qualitative variables were analysed using the relevant chi-square or Fisher's exact test. A p value of <0.05 was regarded as statistically significant, with a highly significant p value of <0.001. The data were input into an MS Excel spreadsheet (Microsoft Corporation, Redmond, WA) and analysed using Statistical Package for Social Sciences version 23.0 (IBM Corp., Armonk, NY).

## Results

**Figure 1:**
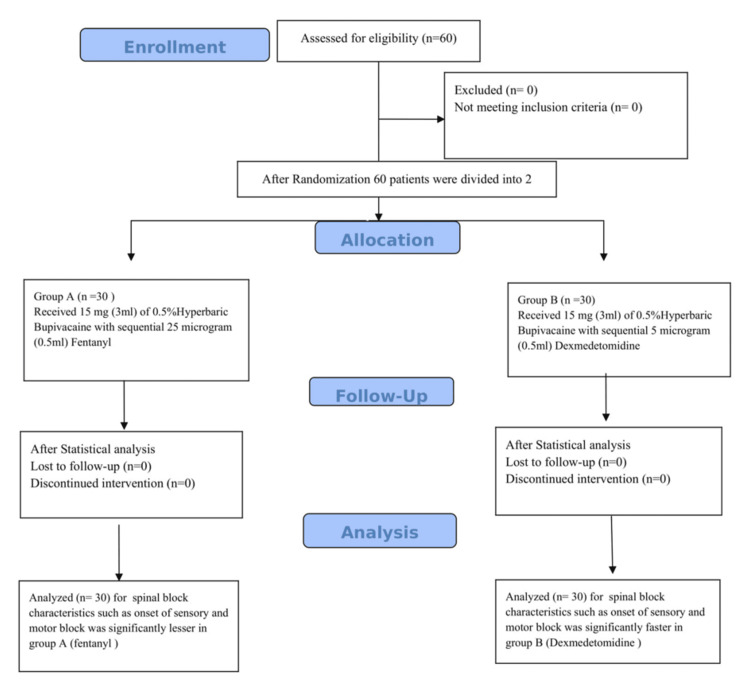
CONSORT flow diagram CONSORT: Consolidated Standards of Reporting Trails

This double-blind, prospective randomised study was conducted in the Department of Anaesthesiology at the Uttar Pradesh University of Medical Sciences, aiming to assess the effects of dexmedetomidine and fentanyl as sequential adjuvants with intrathecal hyperbaric bupivacaine (0.5%) in patients scheduled for elective lower limb surgeries (Figure 1). The primary objectives of the study were to evaluate and compare the onset of sensory and motor block and regression time and assess postoperative analgesic demands through VAS scores and haemodynamic stability among the groups provided by these adjuvants.

 Demographic parameters were comparable between both groups (Table 1).

**Table 1:** Demographic parameters SD: standard deviation; BMI: body mass index; ASA PS: American Society of Anaesthesiologists physical status A p value of >0.05 is considered nonsignificant

Parameters	Group A (n = 30), mean ± SD	Group B (n = 30), mean ± SD	p value
Age (years)	35.03 ± 12.13	37.77 ± 10.44	0.352
BMI (kg/m^2^)	22.83 ± 1.94	22.32 ± 3.27	0.233
Gender (M/F)	27/3	25/5	0.448
ASA PS class I/II	16/14	14/16	0.626
Duration of surgery (minutes)	94.23 ± 8.08	93.33 ± 11.60	0.302

Table 2 shows the block characteristics of group A and group B during the study period. The difference in the mean value in both groups was highly significant (p < 0.001).

**Table 2:** Characteristics of the block SD: standard deviation A p value of <0.001 is considered highly significant

Block characteristics (minutes)	Group A (n = 30), mean ± SD	Group B (n = 30), mean ± SD	p value
Time taken for maximum sensory blockade to T6	5.03 ± 0.81	2.40 ± 0.50	<0.001
Time to two-segment regression	116.53 ± 11.52	161.77 ± 11.88	<0.001
Time to sensory regression to S1	146.57 ± 19.01	207.33 ± 20.18	<0.001
Onset of modified Bromage 3	7.03 ± 0.81	4.23 ± 0.68	<0.001
Regression to modified Bromage 0	169.37 ± 21.66	238.97 ± 19.16	<0.001

Table 3 shows the time to first rescue analgesia, the amount of analgesic administered, and patient satisfaction scores both immediately after surgery and 12 hours into the postoperative period. The difference in the mean value in both groups was highly significant (p < 0.001).

**Table 3:** Comparison of rescue analgesia and patients satisfaction score SD: standard deviation A p value of <0.001 is considered highly significant

Parameters	Group A (n = 30), mean ± SD	Group B (n = 30), mean ± SD	t value	p value
Time to first rescue analgesia requirement (minutes)	259.87 ± 23.72	385.67 ± 22.66	-21.006	<0.001
Total amount of analgesic administered (mg)	225.00 ± 0.00	125.00 ± 35.96	15.232	<0.001
Patient satisfaction scores				
Immediately after surgery	6.00 ± 0.00	7.00 ± 0.00	-	<0.001
Twelve hours following surgery	5.00 ± 0.00	6.00 ± 0.00	-	<0.001

There were statistically significant differences in mean VAS scores in both groups until 340 minutes during the postoperative period (p < 0.001) (Table 4).

**Table 4:** Comparison of VAS postoperatively between the groups VAS: visual analogue scale; SD: standard deviation A p value of <0.001 is considered highly significant

Time interval	VAS score post-op, mean ± SD		z value	p value
	Group A (n = 30)	Group B (n = 30)		
At 30 minutes	0.23 ± 0.43	0.03 ± 0.18	1.000	0.024
At 60 minutes	1.50 ± 0.78	0.23 ± 0.50	7.492	<0.001
At 120 minutes	2.63 ± 0.81	1.60 ± 0.77	5.068	<0.001
At 240 minutes	4.13 ± 1.01	2.47 ± 0.82	7.028	<0.001
At 360 minutes	5.53 ± 0.82	3.73 ± 0.98	7.717	<0.001
At 720 minutes	6.67 ± 0.84	6.30 ± 0.95	3.447	0.120
At 1,440 minutes	7.77 ± 0.63	7.43 ± 1.22	1.329	0.189
24 hours after surgery	7.03 ± 0.93	6.83 ± 0.83	11.728	0.384

There were no notable statistical differences in haemodynamic parameters between the groups. Both groups maintained stable haemodynamics both intraoperatively and postoperatively (Table 5).

**Table 5:** Comparison of haemodynamic parameters among both groups SD: standard deviation A p value of <0.05 is considered significant

Time interval	Haemodynamic parameters	Group A, mean ± SD	Group B, mean ± SD	t value	p value
0 minutes	Heart rate	93.03 ± 5.67	93.43 ± 4.73	-0.297	0.768
	Systolic blood pressure (mm Hg)	124.37 ± 4.04	123.33 ± 3.45	1.066	0.291
	Diastolic blood pressure (mm Hg)	77.50 ± 6.24	80.07 ± 6.47	-1.564	0.123
5 minutes	Heart rate	89.47 ± 4.30	88.80 ± 3.70	0.644	0.522
	Systolic blood pressure (mm Hg)	120.63 ± 4.30	122.00 ± 4.87	-1.152	0.254
	Diastolic blood pressure (mm Hg)	74.33 ± 6.25	76.90 ± 6.09	-1.611	0.113
15 minutes	Heart rate	85.10 ± 5.63	82.57 ± 4.92	3.087	0.069
	Systolic blood pressure (mm Hg)	114.77 ± 7.19	117.37 ± 9.33	-1.209	0.231
	Diastolic blood pressure (mm Hg)	71.43 ± 6.39	70.33 ± 6.88	0.642	0.524
30 minutes	Heart rate	77.37 ± 5.80	74.53 ± 5.5	3.288	0.086
	Systolic blood pressure (mm Hg)	109.10 ± 10	110.13 ± 8.47	-0.496	0.622
	Diastolic blood pressure (mm Hg)	66.87 ± 4.67	65.10 ± 5.86	1.292	0.201
45 minutes	Heart rate	75.37 ± 7.22	73.67 ± 6.02	3.322	0.326
	Systolic blood pressure (mm Hg)	106.53 ± 6.10	107.37 ± 7.06	-0.489	0.627
	Diastolic blood pressure (mm Hg)	65.63 ± 5.46	64.13 ± 5.47	1.063	0.292
1 hour	Heart rate	73.27 ± 6.56	70.57 ± 5.30	4.351	0.085
	Systolic blood pressure (mm Hg)	104.77 ± 5.63	103.33 ± 7.13	0.864	0.391
	Diastolic blood pressure (mm Hg)	64.27 ± 5.55	62.63 ± 5.04	1.193	0.238
2 hours	Heart rate	65.50 ± 3.54	65.75 ± 2.06	-0.115	0.914
	Systolic blood pressure (mm Hg)	104.50 ± 3.54	109.25 ± 9.22	-0.671	0.539
	Diastolic blood pressure (mm Hg)	62.00 ± 4.24	62.25 ± 4.50	-0.065	0.951

The occurrence of nausea was more frequent in group A (fentanyl) compared to group B (dexmedetomidine), although the difference between the groups was not statistically significant (p > 0.05). In group B (dexmedetomidine), five patients experienced bradycardia, which was treated with an injection of atropine (0.6 mg intravenous). Notably, significant side effects like vomiting, tachycardia, hypertension, and itching were not observed.

## Discussion

Dexmedetomidine is a highly selective α2 adrenoceptor agonist that acts as a central sympatholytic, blocking the release of noradrenaline by binding to presynaptic C fibres. This action leads to a decrease in the release of neurotransmitters, results in hyperpolarisation of postsynaptic dorsal horn neurons, and reduces the incidence of shivering and postoperative analgesic requirement. The optimum subarachnoid dosage of 5 mcg enhances the onset of the block, extends its duration, and diminishes postoperative analgesic needs. Fentanyl is a lipophilic µ-opioid receptor agonist. Fentanyl exerts its effects intrathecally via binding to opioid receptors in the dorsal horn of the spinal cord, potentially resulting in supraspinal dissemination and action. Incorporating an adjuvant into a hyperbaric local anaesthetic may modify its density and affect its distribution to the intrathecal space. Consistent with our research, Paramasivan et al. [1] found that patients administered intrathecal dexmedetomidine had reduced VAS scores at 24 hours postoperatively compared to those given a placebo, with a mean difference (95% confidence interval) of -1.05 (-1.89 to -0.20), p < 0.05. Both groups had steady haemodynamics during the intraoperative and postoperative periods. A study conducted by Kalbande et al. [3] yielded the same results, revealing that the duration required to achieve maximum sensory blockage (T6) was markedly reduced in group BD (3.11 ± 0.43 minutes) in contrast to group BF (5.55 ± 0.60 minutes). The current investigation revealed that the highest sensory level attained was comparable in both groups, specifically at the T6 dermatome; however, group B reached this level quicker than group A. It offers a distinct benefit, as achieving the sensory block at the T6 dermatome earlier allows for the commencement of surgery sooner in group B than in group A. The two-segment regression of sensory block and the duration for regression of sensory block to the S1 dermatome were prolonged by injecting intrathecal hyperbaric bupivacaine in conjunction with successive dexmedetomidine.

Imbelloni et al. [14] conducted a study on the impact of incorporating adjuvants into intrathecal local anaesthetics, discovering that the addition of adjuvants may modify the solution's density at 37°C, but the resulting solution remained hyperbaric.

Desai et al. [15] demonstrated that the combination of intrathecal opioids with hyperbaric bupivacaine for intrathecal administration led to an increased opioid requirement during the postoperative phase. They concluded that both hyperbaric bupivacaine and opioids achieve optimal efficacy at their original densities, and their mixture alters these densities, influencing distribution in the cerebrospinal fluid and consequently diminishing the duration of analgesia. Consequently, sequential delivery preserves the physical characteristics of both medications, yielding maximum results. The current study found both groups to be comparable regarding demographic profile, duration and kind of operation, and baseline haemodynamic parameters. In group B, the duration to attain maximum sensory blockade (T6) was shorter, whereas the regression of sensory block by two segments and the regression to the S1 dermatome was delayed compared to group A. Consistent with our research, Mahendru et al. [16] reported that the average duration for two-segment sensory block regression was 147 ± 21 minutes in the dexmedetomidine group, 117 ± 22 minutes in the clonidine group, 119 ± 23 minutes in the fentanyl group, and 102 ± 17 minutes in the normal saline group (p < 0.001). Al-Ghanem et al. [17] observed that the duration to attain the S1 segment was markedly prolonged in the dexmedetomidine group (274.8 ± 73.4 minutes) relative to the fentanyl group (179.5 ± 47.4 minutes). The duration to attain a total motor block was dramatically reduced in group B (dexmedetomidine) relative to group A (fentanyl) (p < 0.001). The extension of a motor block from spinal anaesthetics may occur due to the binding of α2 adrenoceptor agonists to motor neurones in the dorsal horn. Chaudhry et al. [18] indicated that patients in the sequential group (S) attained Modified Bromage 3 more rapidly (6.1 ± 1.296 minutes) compared to those in the premixed group (P) (7.5 ± 1.333 minutes) (p < 0.001). The reduced duration to attain Modified Bromage 3 in our trial may be ascribed to the elevated dosage of bupivacaine (15 mg) administered, in contrast to the 12.5 mg utilised in their investigation. The duration of regression of motor blockage to Modified Bromage 0 was prolonged in group B relative to group A. Suthar et al. [19] noted that the duration of regression of motor block to Bromage 0 was prolonged in the dexmedetomidine group compared to both the clonidine group and the bupivacaine-only group. Group B had an extended duration of analgesia relative to group A. The progressive addition of dexmedetomidine to intrathecal hyperbaric bupivacaine resulted in extended postoperative analgesia and a reduced need for rescue analgesia during the postoperative period. The average VAS scores were reduced in the dexmedetomidine group relative to the fentanyl group. Singam and Mankhair [20] concluded that the two-syringe technique of fentanyl and hyperbaric bupivacaine makes a significant improvement in the quality of sensory blockade with relatively less occurrence of hypotension in patients compared with the one-syringe technique. These findings are consistent with our study. Gupta et al. [21] performed a comparative analysis of intrathecal dexmedetomidine and fentanyl as adjuncts to bupivacaine, noting that patients in both cohorts maintained haemodynamic stability. The occurrence of side effects and problems was analogous between the two groups, as indicated by prior investigations. The principal weakness of this study was its concentration on relatively healthy adults, which complicates the extrapolation of the findings to older patients with cardiovascular comorbidities. A further disadvantage was the inability to ascertain critical outcomes, including chronic pain, long-term safety profiles, and the possibility of neurotoxicity linked to the intrathecal administration of dexmedetomidine. Finally, the subarachnoid block was administered when the patient was seated, and the subsequent shift to a supine position may affect the drug's flow dynamics in the cerebrospinal fluid, influencing the block's properties.

## Conclusions

The study finds that administering dexmedetomidine sequentially as an adjuvant with 0.5% hyperbaric bupivacaine, a subarachnoid block, speeds up the onset of sensory and motor block, offers prolonged analgesia, decreases overall analgesic needs, and maintains stable haemodynamics, all without significant side effects compared to fentanyl for the elective lower limb surgeries. Patient satisfaction scores, immediately after the surgery and 12 hours following surgery, are significantly better in dexmedetomidine than in the sequential fentanyl group. A few patients in the dexmedetomidine group had bradycardia, while nausea was more prevalent in the fentanyl group compared to the dexmedetomidine group, although this difference was not statistically significant.
